# Investigation of Attentional Bias in Obsessive Compulsive Disorder with and without Depression in Visual Search

**DOI:** 10.1371/journal.pone.0080118

**Published:** 2013-11-08

**Authors:** Sharon Morein-Zamir, Martina Papmeyer, Alice Durieux, Naomi A. Fineberg, Barbara J. Sahakian, Trevor W. Robbins

**Affiliations:** 1 Department of Psychiatry, University of Cambridge School of Clinical Medicine, Addenbrooke’s Hospital, Cambridge, United Kingdom; 2 Behavioural and Clinical Neuroscience Institute (BCNI), University of Cambridge, Cambridge, United Kingdom; 3 Division of Psychiatry, University of Edinburgh, Edinburgh, United Kingdom; 4 Department of Psychiatry, Queen Elizabeth II Hospital, Welwyn Garden City, Hertfordshire, United Kingdom; 5 Postgraduate Medical School, University of Hertfordshire, Hatfield, United Kingdom; 6 Department of Psychology, University of Cambridge, Cambridge, United Kingdom; Institute of Psychiatry at the Federal University of Rio de Janeiro, Brazil

## Abstract

Whether Obsessive Compulsive Disorder (OCD) is associated with an increased attentional bias to emotive stimuli remains controversial. Additionally, it is unclear whether comorbid depression modulates abnormal emotional processing in OCD. This study examined attentional bias to OC-relevant scenes using a visual search task. Controls, non-depressed and depressed OCD patients searched for their personally selected positive images amongst their negative distractors, and vice versa. Whilst the OCD groups were slower than healthy individuals in rating the images, there were no group differences in the magnitude of negative bias to concern-related scenes. A second experiment employing a common set of images replicated the results on an additional sample of OCD patients. Although there was a larger bias to negative OC-related images without pre-exposure overall, no group differences in attentional bias were observed. However, OCD patients subsequently rated the images more slowly and more negatively, again suggesting post-attentional processing abnormalities. The results argue against a robust attentional bias in OCD patients, regardless of their depression status and speak to generalized difficulties disengaging from negative valence stimuli. Rather, post-attentional processing abnormalities may account for differences in emotional processing in OCD.

## Introduction

Abnormal affective processing is central to both anxiety and depressive disorders [1–3]. Adopting a processing bias for concern-related stimuli may contribute to vulnerability or maintenance factors in clinical anxiety states [2,4]. Numerous studies have shown greater attentional biases for negative or threat stimuli in depressed and anxious patients [5–7]. For instance, relative to controls, in the emotional Stroop task, anxiety patients were slower color naming words depicting threat than neutral content [8]. Similarly, such patients show attentional bias toward mood-congruent and concern-related material over and above the levels displayed by normal volunteers [9], as in the dot-probe task when responding to targets that follow threat rather than neutral cues [10].

Cognitive theories suggest obsessive compulsive disorder (OCD) should similarly feature abnormal attentional processing towards concern-related material [11,12]. Namely, processing biases in OCD would be expected to contribute to the development and maintenance of intrusive obsessive thoughts. OCD is characterised by obsessions, recurrent intrusive thoughts, and/or compulsions, ritualistic repetitive behaviours or mental acts (American Psychiatric Association; APA, 2000). The content and form of obsessions and compulsions are highly idiosyncratic varying widely across individuals. Research thus far has yielded conflicting findings regarding attentional biases in OCD, which appears anomalous compared to other anxiety disorders [13]. Using the emotional Stroop, several studies found increased interference in OCD patients [14–17], however many more have failed to replicate this [18–25]. Use of the dot-probe, spatial cuing and similar tasks has been similarly equivocal with both positive [12,26] and negative [27–29] findings (see Table 1). 

**Table 1:** Summary of previous studies of attentional bias in OCD.

**Study**	**Participants**	**Paradigm**	**Comorbidities**	**No. of medicated individuals**
***Positive patient studies***				
Foa et al. (1993)	23 OCD (washers); 10 OCD (other); 14 HC	Stroop	n/a	n/a
Lavy et al. (1994)	33 OCD; 29 HC	Stroop	n/a	n/a
Tata et al. (1996)	13 OCD; 18 HTA HC; 26 LTA HC	Dot probe	n/a	n/a
Unoki et al. (1999)	14 OCD; 28 HC	Stroop	n/a	12
Moritz et al. (2009)	42 OCD; 31 HC	Spatial cueing	Depression and anxiety disorders	31
Rao et al. (2010)	50 OCD (26 remitted); 50 HC	Stroop	None	44
***Negative patient studies***				
McNally et al. (1992)	24 OCD; 24 PD; 24 HC	Stroop	n/a	n/a
McNally et al. (1994)	16 OCD; 16 PD; 16 HC	Stroop	n/a	n/a
Kyrios & Iob (1998)	15 OCD; 15 HC	Stroop	Depression	n/a
McNeil et al. (1999)	26 OCD; 17 PTSD; 18 MDD	Stroop	None	None
Kampman et al. (2002)	20 OCD; 20 PD, 20HC	Stroop	n/a	n/a
Moritz et al. (2004)	35 OCD; 20 HC	Stroop	Depression and anxiety disorders	16
Van de Heuvel et al. (2005)	16 OCD; 15 PD; 13 Hch; 19 HC	Stroop	None	None
Moritz et al. (2008)	23 OCD; 23 HC	Stroop	Depression and anxiety disorders	15
Moritz & von Muhlenen (2008)	28 OCD; 27 HC	Spatial cueing	Depression and anxiety disorders	22
Harkness et al. (2009)	18 OCD (checkers); 13 HC	Dot probe	n/a	n/a
Siznio da Victoria et al. (2012)	48 OCD; 24 HC	Attentional bias task	n/a	48
***Positive sub-clinical studies^a^***				
Novara & Sanavio (2001)	21 HSHI; 31 LSHI (PI-R, checking subscale)^b^	Stroop	n/a	n/a
Amir at al. (2009)	23 HSHI; 24 LSHI (MOCI)	Dot probe	n/a	n/a
Armstrong et al. (2010)	23 HSHI; LSHI (PI-R, contamination subscale)	Gaze during free viewing	n/a	n/a
Cisler & Olatunji (2010)	23 HSHI; 28 LSHI (PI-R, contamination subscale)	Spatial cueing	n/a	n/a
Armstrong et al. (2012)	19 HSHI; 20 LSHI (PI-R, contamination subscale)	Gaze during free viewing	n/a	n/a

OCD, Obsessive-Compulsive Disorder; HC, healthy control; HTA, High Trait Anxiety; LTA, Low Trait Anxiety; PD, Panic Disorder; PTSD, Post-Traumatic Stress Disorder; Hch, Hypochondriac; HSHI, High Scoring Healthy Individual; LSHI, Low Scoring Healthy Individual; MDD, Major Depressive Disorder; MOCI, Maudsley Obsessive-Compulsive Inventory; PI-R, Padua Inventory-Revised; n/a, not available.

^a^ The following studies were conducted in groups of healthy controls classified according to their score on self-reported scales commonly used to measure obsessive-compulsive symptoms.

^b^ TData are Groups (Subscale) or as otherwise indicated.

Elevated depression levels have been hypothesized to account for some of the discrepancies [19,27] as comorbid depression has been shown to attenuate attentional bias in some anxiety disorders [30], possibly due to its dampening of motivational systems [31]. Comorbid depression is prevalent in OCD, seen in over a third of cases [32], and may have obscured the emotional bias in some individuals [13]. Additional factors such as variance in the degree of personal relevance of the stimuli due to the idiosyncratic nature of the disorder may have also obscured an existing bias [13,24,28]. Personal relevance has been shown to lead to greater emotional Stroop interference [33]. Hence, experimenter-determined stimuli may not be relevant for individual patients. The extent of attentional biases in OCD has implications for theoretical accounts of OCD, its treatment and nosology [13,34].

 Attention-related processing biases can also be investigated in tasks where participants search for a target amongst distractors [35,36]. Response times are compared for search arrays of different sizes, in which the identity of target and distractor categories is exchanged. This method, where the target is of immediate relevance to the participants’ goal, has proven particularly useful for studying attention with concern-specific pictorial cues [35]. Visual search allows for the investigation of (a) facilitated detection, whereby negative stimuli draw attention towards themselves yielding faster responses; and (b) disengagement difficulties from negative distractors [36,38]. Faster responses in target present displays, likely indicative of facilitated detection (though see [37]), imply increased attention to particular stimuli which may even be enhanced for personal concern-relevant targets. Such a bias may be adaptive in nature [36]. Thus, latencies are faster for detecting negative or concern-related stimuli, with shallower search slopes as set size increases [39,40]. Difficulties in attentional disengagement can be observed particularly in target absent trials, where attention holding components result in longer latencies with the presentation of concern-related stimuli [35,38,41]. A theoretical framework has proposed an evolved fear module based on the concept of biological preparedness, but it has since been suggested that general fear relevance is an important determinant of attention [36,38].

 The present study examined whether individuals with OCD would demonstrate an abnormal processing bias in visual search. This could provide additional measures of concern-related biases as reservations have been raised about the appropriateness and psychometric properties of the emotional Stroop and dot-probe paradigms [42–44]. Moreover, the use of converging methodology with a rich background in anxiety research could potentially shed light on the inconsistencies in the literature. 

## Experiment 1

This experiment investigated abnormal processing bias for images depicting OCD-relevant materials in non-depressed and depressed OCD patients. Depressed OCD patients were included separately to verify whether this factor influenced affective biases in OCD [19]. Images were employed to avoid potential confounds of subjective familiarity and to ensure compelling stimuli [23,45]. To address the idiosyncratic nature of concerns reported by patients [24,28], all participants first rated images of OCD concern-related scenes. An alternative would be to derive personally relevant stimuli for patients and then yoke individual controls [46]. However, this leads to group differences in familiarity and reported personal relevance. Ensuring that a person’s set of most unpleasant scenes was selected, addressed previous concerns that nominally threatening stimuli could be functionally neutral for certain patients [12,20]. Following the rating task, participants searched for their most negative images amongst their positive images and vice versa. 

### Methods

#### Participants

Participants were 36 patients with OCD, and 18 age, gender and verbal IQ matched healthy control participants. Patients were recruited from a specialist OCD outpatient centre and were all diagnosed with OCD by a consultant psychiatrist (NF) according to DSM-IV criteria [47] and an extended clinical interview supplemented by the Mini-International Neuropsychiatric Inventory (MINI; [48]). Though comorbid anxiety and depression symptoms were not excluded provided OCD was the principal diagnosis, patients with other DSM-IV Axis-I comorbidities were excluded as were patients with a history of head injury or other neurological, developmental or medically relevant disorders. Patients were divided into depressed and non-depressed based on their scores on the Montgomery-Asberg Depression Rating Scale (MADRS; [49]). There was a maximal MADRS cut-off of 10 for non-depressed patients and controls and a minimal cut-off of 20 for depressed patients. Seventeen OCD patients with depression were prescribed selective serotonin reuptake inhibitors (SSRIs) of which one was also prescribed a low dose of atypical neuroleptic and one patient was medication free. Of the non-depressed OCD one was medication free and the remaining 17 were prescribed SSRIs, 7 of whom were also prescribed a low dose of an atypical neuroleptic. Controls were recruited via advertisements and were screened for the exclusion criteria of present or past psychiatric illness, history of head injury or neurological disorder and psychotropic medication. Participants were compensated at £8 an hour for their time. 

#### Materials

Current OCD symptom severity was assessed by the Yale-Brown Obsessive Compulsive Scale (YBOCS; [50]) and the Padua Inventory-Revised (PI-R;[51]). The YBOCS is an interview-based scale, with items 1-5 pertaining to obsession severity and items 6-10 pertaining to compulsion severity. The self-report PI-R assesses consists of 39 items which individuals endorse on a five-point scale. Depression severity was assessed by the MADRS [49], which is a 10-item interview-based questionnaire. Finally, current and long-lasting anxiety levels were assessed by the State-Trait Anxiety Inventory (STAI; [52]).

The stimuli consisted of 100 images obtained from various stock photography websites. To avoid the confound of presenting images of different categories [53], all images depicted scenes from around the house with no people. Five clinicians and OCD researchers rated an initial set of 160 images for OCD relevance. The final 100 images were further screened for uniform quality and complexity, and where necessary adjusted for colour saturation and proportion. Some images depicted aesthetic, neat or symmetrical scenes, while others depicted a wide variety of OCD-relevant concerns. Images included mess, clutter, and dirt encompassing toilets, sinks, doors, shelves, cleaning materials, bathrooms, kitchens, dining rooms, home-offices and bedrooms. Additional scenes depicted dangerous situations (e.g., knife in toaster, open gas tops, overloaded electrical sockets), or unpleasant events (e.g., car with crashed front, broken-into front door, knife with blood). There were also potentially neutral images such as light switches, receipts and stationary. There were at least 12 images available for common themes such as contamination, harm avoidance, hoarding and symmetry/ordering. Four mask collage images for the search task were created from 40 additional images, with each mask composed of at least 10 images. Images were 200 by 150 pixels in size, and were presented against a grey background. The tasks were programmed using Visual Basic .NET and were presented on a Paceblade Avantech Slimbook A110. Responses were made with a standard mouse for the rating task and a custom button-box for the search task.

#### Procedure

The study was approved by the local research ethics committee (Bedforshire REC; 07/Q0202/10)) and all participants provided written, informed consent before testing. Participants first rated each of the 100 images on a 7-point Likert scale ranging from “very unpleasant” to “very pleasant”. The images were presented individually in random order, the pace was self-determined and participants were encouraged to use the entire range. On completion, the 12 most positive and 12 most negative images were automatically selected and used in the search task. In the search task, participants searched for a target image in a display of 4 or 12 images arranged in a 4x4 grid. The locations in each display were randomly determined so that in the 4-image displays, each image appeared in a separate row and column, and in the 12-image displays there was exactly one blank in each row and column. On each trial a fixation cross appeared for 500 milliseconds (ms), followed by the target probe (1500 ms, 85% in size) that was covered by a randomly chosen mask (500 ms), then a subsequent fixation (500 ms), which was replaced by the search display. A response terminated the display and was followed by a 1000 ms inter-trial interval. Employing a target prime has been successfully employed in studies of generalized anxiety disorders, depression and social phobia [54,55] and ensured low error rates. The visual mask and 1000 ms delay reduced the likelihood that the guided search would rely on low-level visual memory. 

Participants indicated whether the target image was present or absent by pressing left and right keys, with the mapping counterbalanced within group. Participants were instructed to respond as quickly and as accurately as possible. A positive target was presented against a background of negative distractors and vice versa. There were 192 trials, with 24 trials in each of the eight conditions (target presence x target/distractor category x display size). Stimulus location was randomly determined though all columns and rows were employed equally in each display. The task was preceded by 12 practice trials using a separate set of neutral images, and there were breaks every 48 trials. Trial order was random, with the constraint that 12 exemplars of each display type were presented in each half. Each image appeared 4 times as a target, and distractors were randomly chosen for each display. 

#### Data Analysis

Mean valence rating scores and reaction times (RTs) were calculated for the rating task, and correct mean RT and accuracy were calculated for the search task. Search trials with RTs slower than 4500 ms, consisting of 0.8% of trials, were omitted from the analyses. Likewise, as the rating task did not have practice trials, the first three trials did not contribute to mean RT calculation to avoid outliers. Analyses of variance (ANOVA) were conducted with simple effects following significant interactions and Tukey’s honest significant different (HSD) for post-hoc pairwise comparisons where appropriate. Analyses were conducted using STATISTICA 8.0 software (StatSoft, Inc., Tulsa, OK).

In the rating task the design included Group (depressed OCD/ non depressed OCD/ controls) and where appropriate valance rating (1-7). The Huynh-Feldt correction (ε) was employed when sphericity was violated for valence rating. Following previous studies [36], separate ANOVAs were performed in the search task for RTs to displays with and without targets. The design included Group (depressed OCD/ non depressed OCD/ controls) x Image category (target negative/positive) x Set-size (4/12). To complement these analyses, search slopes were calculated by dividing the overall increase in RT by the number of additional distractors in the display. Pearson Correlation Coefficients were used for correlation analyses between questionnaire scores and task performance, and Cohen’s d was calculated for between group differences of interest.

### Results

#### Demographic and Clinical Characteristics

As seen in Table 2 non-depressed OCD, depressed OCD and control participants did not differ in age, verbal intelligence as measured by the National Adult Reading Test (NART;[56]), or gender distribution, (*ps*>.50). One-way ANOVAs comparing the three groups revealed significant differences on OCD symptom severity, depression and anxiety (ps<.01). Post-hoc Tukey comparisons confirmed that both OCD groups scored higher than controls on the YBOCS, Padua and trait anxiety (*ps*<.01). Depressed OCD group scored higher on the MADRS than both the non-depressed OCD group and controls, (*ps*<.001), which did not significantly differ (*p*=.30). The two OCD groups did not differ on the YBOCS (*p*=.39) or state anxiety (*p*=.23). They did however differ in the self-rated PI-R, (*p*<.001), and non-depressed had marginally lower trait anxiety scores (*p*=.09).

**Table 2:** Participant characteristics and rating data for Experiment 1.

**Characteristics**	**Clinical relevance**	**OCD depressed**	**OCD non-depressed**	**Control**	**Statistic**
*N*		18	18	18	
Gender (M:F)		10:8	9:9	9:9	
Age (y)		41.61^a^ (13.55)	40.72^a^ (13.31)	40.72^a^ (11.73)	*F*<1
NART	Verbal IQ	116.06^a^ (6.51)	116.67^a^ (7.55)	115.39^a^ (6.90)	*F*<1
YBOCS	OCD severity	24.33^a^ (6.24)	21.72^a^ (8.17)	0.67^b^ (1.14)	*F*=83.72
MADRS	Depression	24.67^a^ (6.97)	7.44^b^ (5.50)	4.72^b^ (3.56)	*F*=37.59
STAI-S	State Anxiety	44.72^a^ (11.08)	39.44^a^,^b^ (10.51)	31.89^b^ (6.21)	*F*=9.62
STAI-T	Trait Anxiety	61.67^a^ (11.0)	55.11^a^ (8.87)	37.56^b^ (7.47)	*F*=94.65
PI-R	OCD severity	63.44^a^ (21.26)	42.56^b^ (15.03)	11.44^c^ (6.55)	*F*=27.18
COWC	Contamination obsessions and washing compulsions	19.22^a^ (9.66)	14.61^a^ (9.27)	3.89^b^ (3.39)	*F*=17.51
DRGRC	Dressing/grooming compulsions	6.28^a^ (4.51)	4.06^a^ (3.83)	0.89^b^ (1.18)	*F*=10.89
CHKC	Checking compulsions	24.11^a^ (8.40)	15.72^b^ (8.44)	4.22^c^ (3.37)	*F*=35.17
OTAHSO	Obsessional thoughts of harm to self/others	10.17 ^a^ (6.08)	5.44 ^b^ (3.09)	1.50 ^c^ (1.65)	*F*=20.64
OITHSO	Obsessional thoughts of harm to self/others	3.67^a^ (4.59)	2.72^a^ (2.95)	0.94^b^ (1.06)	*F*=3.34
Mean Rating		3.66 ^a^ (0.69)	3.59^a^ (0.34)	3.79^a^ (0.36)	*F*=1.01
Mean Rating RT (ms)		4831^a^ (2049)	4917^a^ (1504)	3339^b^ (1058)	*F*=5.61

Values are mean (standard deviation) or as otherwise indicated.

M, male; F, female; IQ, intelligence quotient; YBOCS, Yale-Brown Obsessive-Compulsive Scale; MADRS, Montgomery-Asberg Depression Rating Scale; STAI, State-Trait Anxiety Inventory; - S, state; - T, trait; PI-R, Padua Inventory-Revised; RT, Reaction Time.

Values on the same line sharing the same superscript are not significantly different as assessed by Tukey post-hoc comparisons.

#### Visual Ratings

**Figure 1:**
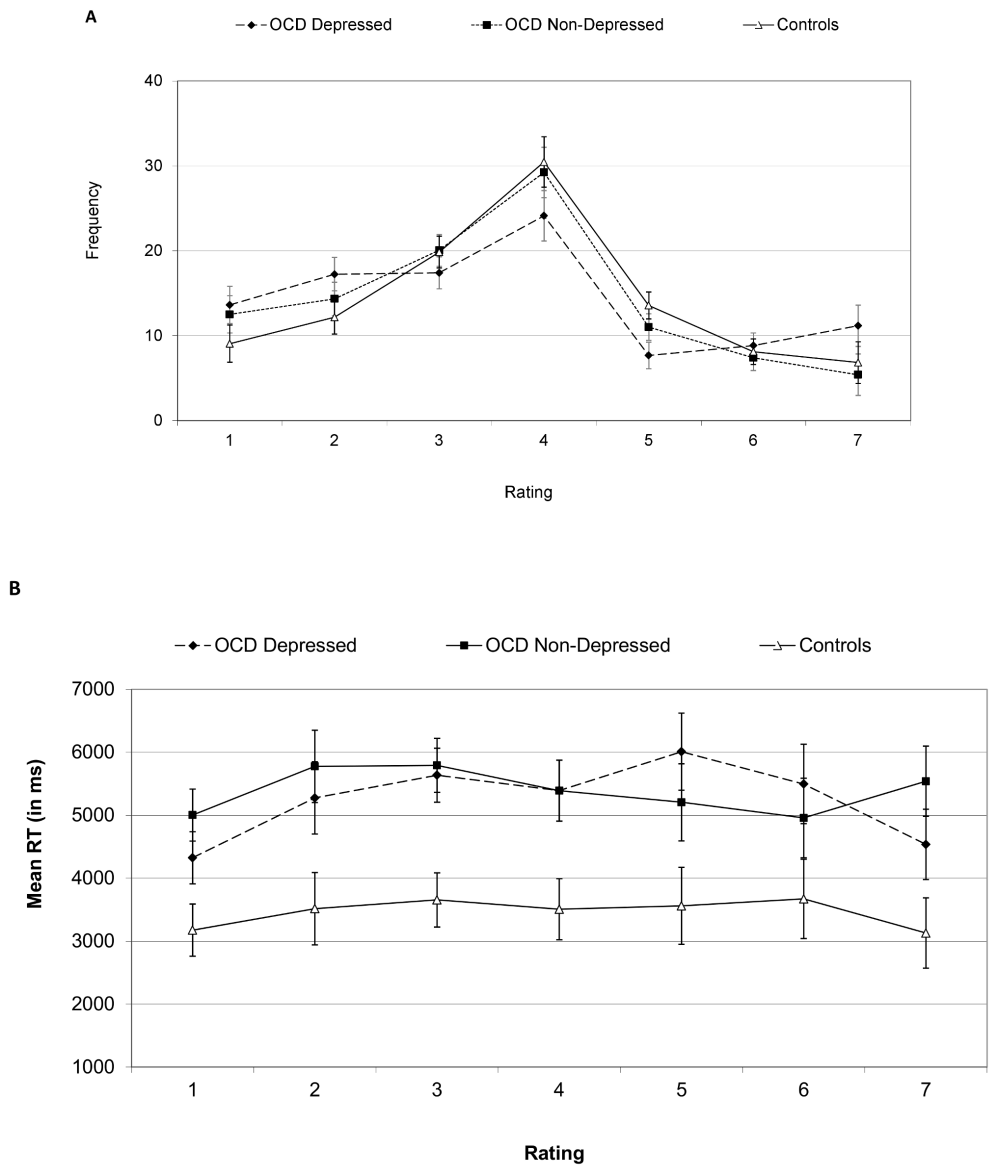
Mean frequency of responses (A) and reaction time (B) in Experiment 1 rating task. Ratings 1, 4 and 7 denote very unpleasant, neutral and very pleasant valence, respectively. Error bars are one standard error of the mean values. OCD, Obsessive Compulsive Disorder.

As seen in Table 2, one-way ANOVAs on the ratings and their RTs indicated no significant group differences in mean valence rating, (*p*>.35) but an effect of group on RTs (*p*<.01). Post-hoc Tukey comparisons indicated both OCD groups were slower than controls (*ps*<.05, d=0.91, and d=1.21, for depressed and non-depressed groups, respectively), but did not differ from each other (*p*>.5). Secondary two-way ANOVAs on image frequency with rating score values (1-7) and group as independent factors revealed an effect of rating score, *F*(6, 306)=29.21, *p*<.01, ε=0.78, with no interaction between rating values and group, *p*>.17. Likewise, there was an effect of rating values on RTs, *F*(6, 306)=2.36, *p*<.05, ε=0.79, due to faster latencies when rating images as more extreme (negative or positive), with a significant quadratic contrast, *F*(1, 51)=7.45, *p*<.01. There was no interaction between rating values and group on RTs, *p*>.40 (see Figure 1). In sum, though there were no significant group differences in rating scores, OCD patients were slower to rate the images than controls, regardless of depression status.

#### Visual Search

Accuracy was high (96%) and as analyses indicated no effects for group, *p*>.50, this measure was not analyzed further. RT analyses revealed effects for block in both target present and target absent trials, *F*(1, 51)=40.5, *p*<.01, and *F*(1, 51)=17.09, *p*<.01, respectively. RTs were faster in the second block (1127 vs. 1228 ms, and 1857 vs. 1959 ms for target present and absent displays, respectively). As block did not interact with any factor, the data were collapsed across block. 

**Figure 2:**
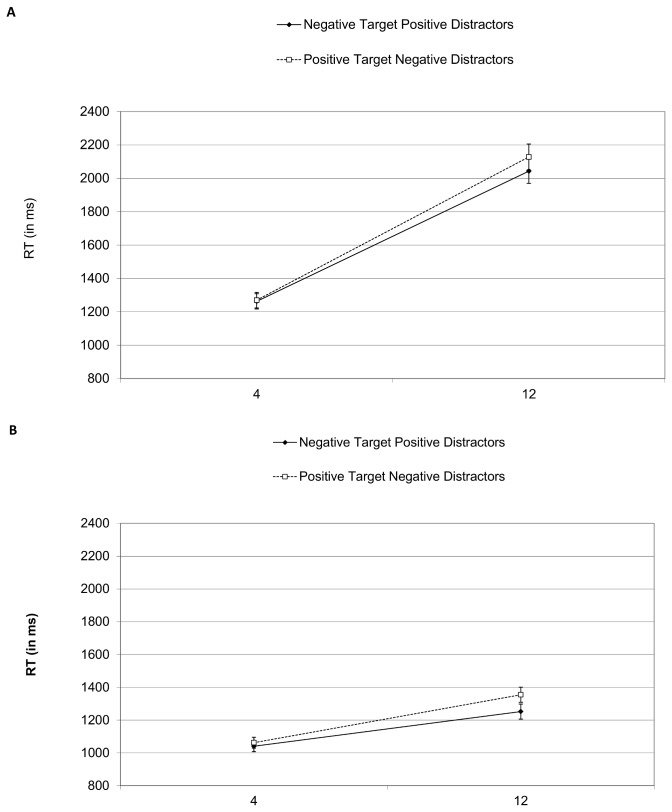
Mean reaction times for target absent (A) and target present (B) trials in Experiment 1. Error bars are one standard error of the mean values.

Figure 2 presents search RTs in target absent and present displays. The ANOVA for target present displays included group, image category and set-size as factors and indicated marginally slower latencies for depressed OCD (1162 ms) compared to non-depressed OCD (1018 ms) and controls (962 ms), *F*(2, 51)=2.55, *p*<.09. Post-hoc Tukey comparisons showed a marginal difference between controls and depressed OCD (*p*<.09) with the non-depressed not significantly differing from either group (*p*>.25 for both comparisons). There were main effects for image category, *F*(1, 51)=11.53, *p*<.01, and set-size, *F*(1, 51)=168.04, *p*<.01,and an interaction between them, *F*(1, 51)=9.07, *p*<.01 . The interaction resulted from an effect for image category in the large, *F*(1, 51)=16.28, *p*<.01, but not the small display, *p*>.30.

The corresponding ANOVA on target absent trials revealed slower latencies for depressed OCD (2080 ms) compared to non-depressed OCD (1770 ms) and controls (1769 ms), *F*(2, 51)=3.32, *p*<.05 . Post-hoc comparisons indicated marginal slowing in the depressed OCD (*p*<.08 for both comparisons). There were effects for image category, *F*(1, 51)=5.09, *p*<.05, and set-size, *F*(1, 51)=335.76, *p*<.001, and the interaction, *F*(1, 51)=5.09, *p*<.05, which again resulted from a significant effect for image category in the large, *F*(1, 51)=6.39, *p*<.05, but not the small set-size, *p*>.5. No other effects reached significance (all *p*’s>.25), 

In accordance with these results, an ANOVA on search slopes with group, image category and target status revealed effects for image category, *F*(1, 51)=11.17, *p*<.01, and target status, *F*(1, 51)=199.70, *p*<.001. Search slopes were flatter for negative targets with positive distractors (62 ms) compared to positive targets with negative distractors (72 ms per item). Additionally, slopes were flatter in target-present displays (32 ms per item) vs. target-absent displays (102 ms per item).

In sum, though depressed OCD patients were slower overall to respond, there were no differences in task performance between the groups. For all groups, the search slope was flatter for unpleasant targets. Furthermore, the effect for image category remained in target-absent displays suggesting the unpleasant distractors were slowing the search.

### Discussion

All groups were slower when searching for self-rated positive images amongst negative distractors than amongst negative targets amongst positive distractors. That the effect was found regardless of target presence suggests the attentional bias was largely influenced by disengagement difficulties rather than facilitated detection of positive stimuli [35]. Not only did all groups exhibit a comparable bias, but they also rated the images similarly. Despite markedly different clinical characteristics, performance in the search and rating tasks was similar between the two OCD groups, with only a general slowing noted in the depressed group. Thus, in Experiment 1, the magnitude of attentional bias in the search task was not influenced by the presence OCD or comorbid depression (see also [57]).

At the same time, OCD patients were slower than controls to rate the images. It is possible that a bias was initially present in the patients, but became attenuated over time [58]. To explore this possibility, rating data were analyzed split into 5 blocks of 20 trials. In addition to main effects of group, *F*(2, 51)=5.69, *p*<.01, and block, *F*(4, 204)=15.95, *p*<.01, there was an interaction, *F*(8, 204)=2.82, *p*<.01, whereby both OCD groups became faster over time, whilst the controls did not (linear contrast, *F*(1, 51)=31.09, *p*<.001). Nevertheless, patients remained slower than controls in all blocks. This slowing might have resulted from an attentional bias but other explanations such as general ruminations cannot be excluded.

### Experiment 2

The presence of an attentional bias in healthy volunteers appears inconsistent with some previous studies [36,59]. Given the universal nature of OCD-related concerns, rating the images before the search task may have activated OCD schema in controls and led to an artificially enhanced emotional bias. In the second experiment participants first performed the search task and then rated the images. The search images were identical for all participants and were derived from the most extreme-rated images of Experiment 1. This experiment compared a single group of OCD patients with a control group, given the limited role of depression in Experiment 1. 

### Methods

The methods for Experiment 2 were identical to those of Experiment 1 with the following exceptions.

#### Participants

Participants were 18 patients with OCD and 18 age, gender and verbal IQ matched healthy control participants. Following the results of Experiment 1, the patients were not separated into depressed and non-depressed groups, and participants with a MADRS score between 10 and 20 were not excluded. Sixteen patients were prescribed SSRIs, in 2 supplemented with a low dose of atypical neuroleptics, and 2 patients were medication free. 

#### Procedure

Participants completed the search task, comprising 96 trials, followed by the rating task of the 100 stimuli. The common stimuli set employed in the search task was derived from Experiment 1 ratings. Specifically, across all participants, 12 of the top 13 images (*M*=5.63, *SD*=0.73) and 12 of the bottom 13 (*M*=1.96, *SD*=0.68) were selected ensuring maximal overall visual similarity.

### Results

#### Demographic and Clinical Characteristics

OCD patients and controls did not differ in age, *F*(1, 34)=0.05, *p*>.50, or verbal intelligence, *F*(1, 34)=1.12, *p*>.25. Group differed in OCD, depression and anxiety symptoms severity (*ps*<.001) as presented in Table 3. 

**Table 3:** Participant characteristics and rating data for Experiment 2.

**Characteristics**	**Clinical relevance**	**OCD Patients**	**Controls**	**Statistic**
*N*		18	18	
Gender (M:F)		10:8	10:8	
Age (y)		39.50 (14.04)	40.44 (10.47)	*F*<1
NART	Verbal IQ	119.00 (4.43)	120.44 (3.73)	*F*<1
YBOCS	OCD severity	19.11 (7.45)	0.00 (0.00)	*F*=118.42
MADRS	Depression	14.39 (10.11)	2.28 (2.17)	*F*=24.69
STAI-S	State Anxiety	47.39 (13.41)	31.67 (6.60)	*F*=19.92
STAI-T	Trait Anxiety	56.83 (13.54)	35.28 (7.99)	*F*=33.85
PI-R	OCD severity	53.00 (26.76)	16.50 (17.03)	*F*=23.82
COWC	Contamination obsessions and washing compulsions	18.33(12.00)	5.39 (7.00)	*F*=15.61
DRGRC	Dressing/grooming compulsions	5.33(3.66)	1.72 (1.60)	*F*=14.69
CHKC	Checking compulsions	20.22 (9.98)	6.44 (6.78)	*F*=23.48
OTAHSO	Obsessional thoughts of harm to self/others	7.11 (4.43)	2.33 (2.72)	*F*=15.19
OITHSO	Obsessional thoughts of harm to self/others	2.00 (3.22)	0.611(0.85)	*F*=3.13
Mean Rating		3.53 (0.53)	3.86 (0.31)	*F*=5.17
Mean Rating RT (ms)		3180 (1303)	2502 (531)	*F*=4.18

Values are mean (standard deviation) or as otherwise indicated.

M, male; F, female; IQ, intelligence quotient; YBOCS, Yale-Brown Obsessive-Compulsive Scale; MADRS, Montgomery-Asberg Depression Rating Scale; STAI, State-Trait Anxiety Inventory; - S, state; - T, trait; PI-R, Padua Inventory-Revised ; RT, Reaction Time.

#### Visual Ratings

**Figure 3:**
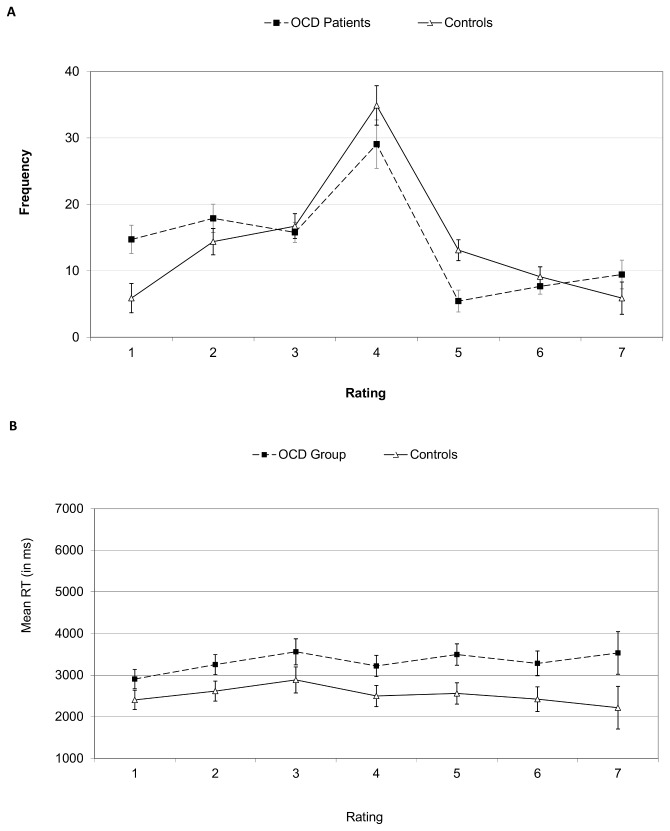
Mean frequency of responses (A) and reaction time (B) in Experiment 2 rating task. Ratings 1, 4 and 7 denote very unpleasant, neutral and very pleasant valence, respectively. Error bars are one standard error of the mean values. OCD, Obsessive Compulsive Disorder.

Figures 3a and 3b depict rating frequencies and RTs for Experiment 2. The OCD group scored the images more negatively, *F*(1, 34)=5.17, *p*<.05, d=0.76, and responded slower, *F*(1, 34)=4.17, *p*<.05, d=0.68, than controls (see Table 3).

Secondary ANOVAs with rating score as a factor indicated an interaction between group and rating, *F*(6, 204)=2.98, *p*<.01, and an effect of rating, *F*(6, 204)=26.38, *p*<.01, ε=0.58. Simple comparisons suggested patients rated more images as 1 (very negative), *F*(1, 34)=8.55, *p*<.01, while controls rated more images as 5 (slightly positive), *F*(1, 34)=10.96, *p*<.001, *ps*>.25 for other ratings). The corresponding ANOVA on RTs showed no significant effect for rating score or the interaction (*p*>.17 for both). In this analysis missing values were replaced by the individual’s mean RT. 

Additional analyses were performed on the image subset used in the search task, with group and image valence (negative/positive) as factors. Negative images scored lower (2.01) than positive images (5.70), *F*(1, 34)=306.16, *p*<.001. OCD patients judged the negative images as more negative than controls, *F*(1, 34)=4.63, *p*<.05, but not the positive images, (*p*>.50). There were no significant differences in rating RTs (*p*>.12 for all).

To examine the time-course of latencies, RTs were analyzed in blocks of 20 trials. Though responses speeded over the 5 blocks, *F*(4, 136)=6.25, *p*<.01, this did not interact with group, *p*>.5, suggesting the OCD group were slower throughout the entire course of the rating task. 

#### Visual Search

**Figure 4:**
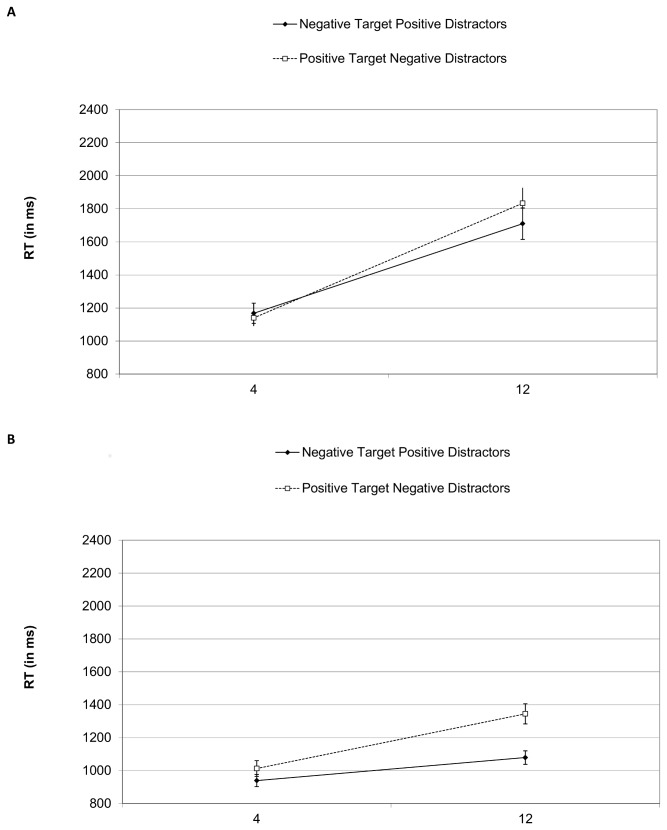
Mean reaction times for target absent (A) and target present (B) trials in Experiment 2. Error bars are one standard error of the mean values.

Figure 4 presents search RTs for target absent and target present trials. Analysis of accuracy revealed no effect for group, *p*>.50, with a mean accuracy of 93%. ANOVAs on target present and absent displays included group, image category and set-size as factors. Results of target present displays revealed significant effects for image category, *F*(1, 34)=44.31, *p*<.01, set-size, *F*(1, 34)=90.93, *p*<.001, and their interaction, *F*(1, 34)=30.24, *p*<.01, resulting from a larger image category effect in the large display, *F*(1, 34)=50.40 , *p*<.001, than the small display, *F*(1, 34)=10.57, *p*<.001). Similar results in the target absent trials showed effects for set-size, *F*(1, 34)=108.96, *p*<.001, and an interaction between image category and set-size, *F*(1, 34)=20.06, *p*<.001, stemming from an effect of image category in the large, *F*(1, 34)=12.53, *p*<.01, but not the small display, *p*>.30.

An ANOVA on search slopes with group, image category and target status revealed significant effects for image category, *F*(1, 34)=48.51, *p*<.001, and target status, *F*(1, 34)=65.51, *p*<.001, and a marginal interaction between group and image category, *F*(1, 34)=3.36, *p*<.08, stemming from a crossover so that slopes were smaller for patients for unpleasant targets in pleasant distractor displays, but larger for positive targets in negative distractors compared to controls. Search slopes were flatter for unpleasant targets with pleasant distractors (43 ms per item) than positive targets with negative distractors (64 ms per item). Likewise, search slopes were flatter when the target was present (30 ms per item) than when it was absent (77 ms per item). 

#### Combined and Correlation Analyses

We examined whether presenting the rating task before the search task influenced the magnitude of the bias by comparing Experiments 1 and 2. Planned comparisons indicated the negative bias was larger in Experiment 2 for large set-size displays, *F*(1, 85)=12.92, *p*<.001, but not small set-size displays, *p*>.5. We also examined correlations between key clinical and task measures in the entire patient sample (see Table 4). Mean image rating was correlated with self-reported OCD symptoms and depression, with lower mood associated with more negative ratings. This was observed also in all the subscales of the Padua, which correlated negatively with mean ratings (*r*=-0.29 to -0.36), with the exception of OITHSO (r=-0.15). In addition to the information provided in Table 4, the Padua subscales did not correlate significantly with any visual search measures (r=-0.14 to r=0.27). Slower ratings were associated with steeper search slopes in all displays, with the exception of negative distractors in target absent displays. Finally, effect sizes for group differences in attentional bias were computed based on all participants. For target present the Cohen’s d was 0.14 and for target absent it was 0.10.

**Table 4:** Correlations between clinical, rating and visual search variables.

**Variable**	**1**	**2**	**3**	**4**	**5**	**6**	**7**	**8**	**9**	**10**
1. YBOCS	-	.34*	.30*	.33*	-.09	.18	.08	.14	.33*	.02
2. MADRS		-	.28*	.62**	-.28*	.15	-.01	.11	.15	.00
3. STAI-T			-	.38*	-.14	-.05	-.19	-.12	-.21	-.16
4. PI-R				-	-.45**	.07	-.02	.12	.00	.17
5. Mean Rating					-	-.27*	.07	.08	.04	-.01
6. Mean Rating RT						-	.32*	.34*	.33*	-.02
7. Search slope: positive distractors no target							-	.34*	.78*	.33*
8. Search slope: negative target								-	.38*	.33*
9. Search slope: negative distractors									-	.44*
10. Search slope: positive target										-

YBOCS, Yale-Brown Obsessive-Compulsive Scale; MADRS, Montgomery-Asberg Depression Rating Scale; STAI, State-Trait Anxiety Inventory; - T, trait; PI-R, Padua Inventory-Revised ; RT, Reaction Time.

* *p*<.05, ** *p*<.01

### Discussion

In agreement with Experiment 1, participants responded slower in the presence of negative distractors than positive distractors. The magnitude of this bias did not significantly differ between the OCD and control groups, again suggesting that while OCD patients demonstrated an emotional bias in the search task, it was not enhanced relative to healthy controls. Experiment 2 indicates that the absence of an enhanced emotional bias in Experiment 1 did not result from its attenuation over time in the rating task, nor was it likely due to priming OCD-relevant concepts or scenes in the healthy controls. Following the search task, OCD participants were slower than controls to rate the images but in contrast to Experiment 1 they also rated them more negatively. This suggests that repeated exposure influenced valence judgments in the OCD participants (see further discussion below). Compared to Experiment 1, the attentional bias was larger and was present in both small and large displays. Importantly though, this did not differ between the groups. 

## General Discussion

This study investigated attentional bias to personally selected concern-related images in OCD patients, with and without depression. OCD and control groups demonstrated similar attentional bias effects for idiosyncratic or common OCD-related images. All groups showed slower latencies to negative than positive images in large displays, and this negative bias was larger without prior exposure. Nevertheless, the OCD groups were slower than controls to rate the images. Following prior exposure to concern-related images, individuals with OCD also rated them as more negative. Negative ratings were associated with increased OC and depressive symptom severity. Taken together the study did not find evidence for an enhanced attentional bias to concern-related materials in OCD. Whilst questioning the functional significance of any abnormal attentional biases in OCD and arguing against their robustness, the results support post-attentional abnormalities in processing emotional content.

### Attentional Bias

The search task revealed slowing with negative distractors regardless of target presence, suggesting difficulties in attention disengagement rather than enhanced threat detection. This is consistent with previous interpretations of Stroop interference and visual search performance that implicate later attentional stages of processing where slowing results from difficulties redirecting attention away from stimuli [35,60]. Slowed disengagement to negative stimuli was not specific to OCD and was noted in the controls, whose demographic and clinical characteristics were well within the normal range. Some previous studies reported reduced or no negative biases in controls [36,59] whilst others reported OCD-related or negative biases [19,24]. The universality of OCD concern themes and the clearly negative valence likely elicited a general bias here. As the bias was observed in Experiment 2, individual image selection could not underlie this finding. Individual image selection or pre-exposure may however, account for a general reduction in the bias in Experiment 1. A broad range of stimuli can lead to disengagement difficulties in healthy individuals, as suggested by results with similar categories, such as faces or animals [38–40]. The attentional processes involved are likely similar with threatening, anxiety provoking, and present negative OCD scenes; supporting the notion of a general mechanism that disrupts on-going strategic attentional processing. Using idiosyncratic stimuli in healthy individuals offers a means with which to explore this further.

There was no evidence for an abnormal attentional bias in OCD: neither with self-selected nor with pre-selected stimuli. Rather, a small effect-size was present in a sizeable sample of chronic symptomatic patients (Cohen’s d<0.15). The second experiment indicated this was unlikely due to attenuation with prior exposure in the OCD group or priming in the controls [61]. Our results, using a search task, are in line with most prior research which failed to find attentional bias differences between OCD and healthy individuals in Stroop, dot-probe or spatial cueing [19–24,27,28]. As previous null findings have typically employed words, it has been suggested that the choice of stimuli may be critical with the need for evocative [28] or attention-grabbing [23] stimuli. Current stimuli were effective in inducing a negative bias, yet we still failed to find an abnormal attentional bias. It could be that the images were too effective at distracting attention (though see 26,60), however given similar ratings in Experiment 1 and the effects on post-attentional processing (see below), a more parsimonious account would be the lack of a reliable and robust enhanced effect in OCD. 

The presence of depression in OCD led to marked clinical differences, particularly in self-report measures, but in accordance with previous findings [27] did not alter the magnitude of attentional bias. Thus, depression is unlikely to have played a significant role in the heterogeneity of previous results [19,62]. OCD patients with depression were slower in the search task across all conditions, though they performed similarly to the non-depressed OCD patients in the rating task. Hence, the slowing may have resulted from task demands, such as maintaining the target in working memory, as short term visual memory and performance on delayed match to sample is impaired in depressed patients [63]. General slowing in the depressed patients is also consistent with temporary freezing of all on-going activity attributable to the presence of threatening stimuli [44],, which presumably could be more easily detected in the speeded search task. It is possible that an attentional bias is only found in a subset of patients with specific symptoms such as contamination or checking [12,29]. However, this hypothesis precludes a dimensional approach to OCD symptoms and in the present study was not supported by correlations between symptom dimensions and attentional bias magnitude, which were uniformly low.

Of note, several studies in subclinical student populations support an abnormal bias across a variety of tasks and measures [58,60,64–66]. These findings generally point to disengagement or late-stage attentional biases, but are mitigated by comparisons with individuals with particularly low OC-related scores, with often limited control for anxiety and depressive severity. Moreover, some have discussed its fragile and transient nature [58]. In any case, without evidence for time-consuming obsessions and compulsions in these student populations, the generalizability of these findings to OCD patients is limited. Our conclusions appear inconsistent with a formal meta-analysis reporting an enhanced attentional bias in OCD with a Cohen’s *d* effect size of 0.45 [34]. However, calculations were based on 6 studies, including one in a subclinical student sample [65] and one with an unusually large effect size (Cohen’s *d*>1.5; [12]). Taken together the data suggest a limited role for attentional bias in maintaining OC symptoms in patients. Compared with other anxiety disorders, any effect would be smaller, more temporary and subtle.

### Post-attentional processing

In line with OCD clinical features, present findings support abnormal post-attentional emotional processing in OCD. First, patients were slower than controls to rate images across all valences. Though this slowing attenuated with time in Experiment 1 likely reflecting habituation [58], it nevertheless remained throughout both experiments. The slowing may relate to judgements involving the self or personal reference, in keeping with the role of personal responsibility [67], and to reported lack of self-confidence and indecisiveness in OCD [62]. Secondly, increased self-reported OC and depression severity were associated with more negative ratings in the patients though both OCD groups were similarly slowed. Thirdly, prior exposure to OCD-related stimuli influenced subjective valence judgements in OCD, with priming of OCD themes in the search task influencing responses to both familiar and novel scenes. These findings dovetail with the suggestion that post-attentional strategic processes rather than attentional mechanisms are more central to the etiology and maintenance of symptoms in OCD [28,62]. This would also be consistent with evidence that individuals with OCD had difficulties in switching away from concern-related but not neutral words [57]. Moreover, although depression plays a limited role in attentional bias in OCD, it may be more central in later stages of processing [9].

### Limitations and future directions

Most individuals with OCD were medicated. Antidepressants have been found to reduce emotional biases to negative facial expressions [68]. Comparable disengagement difficulties across groups may have resulted from normalizing effects of the medication. At the same time, that the patients still suffered marked OCD symptoms argues for a negligible role of attentional biases in symptom maintenance. Moreover, studies with both positive [16,17,26] and negative findings [23,28] employed samples with primarily medicated patients, and at least one study with unmedicated patients reported negative findings [22] indicating this is unlikely to play a determining role. 

The use of images enhanced ecological validity [38], but allowed less control over perceptual features. Search slopes for negative targets were shallower in both experiments, suggesting a more efficient search for negative stimuli, in line with findings that search efficiency depends on meaning and not solely on physical characteristics [39]. Physical characteristics are unlikely to account for the negative bias, as latencies and search slopes support a serial search. Moreover, image selection was remarkably variable, with all images selected for at least one search (*M*=14.16, *SE*=11.10 for number of searches per image). This, together with the randomized selection of distractors and locations render this possibility unlikely. It is also possible that some complex abstract concerns such as fear of becoming an evil person may not be elicited in our study. However, common themes were covered in the stimulus set and crucially, the robust negative bias effect together with the rating values indicates that individuals found the present stimuli aversive. Future studies may associate provocation-related items with abstract stimuli, thus circumventing such concerns [69]. 

Although visual search tasks have been successful in detecting attentional biases in other anxiety disorders, they may not be sufficiently reliable or sensitive in the case of OCD, as appears to be the case for the dot-probe and Stroop tasks. This seems particularly likely for the detection of possible orienting biases [55]. Future studies, employing alternative high temporal resolution measures such as eye movements or event-related potentials, together with symptom-relevant scenes would be particularly useful in patients to establish whether and when biases may be present [70]. Future studies may also explore the possibility that the presence and potential role of attentional biases throughout the course of the disorder may change, with a more prominent role prior to symptom onset, as evidence from the subclinical populations appears to suggest. 
